# Development and analysis of a nano-triangular wave-shaped polarizer

**DOI:** 10.1038/s41598-023-40511-z

**Published:** 2023-08-17

**Authors:** Ryohei Hokari, Kyohei Takakuwa, Kengo Shiomoto, Genki Kuwano, Kazuma Kurihara

**Affiliations:** 1 Advanced Manufacturing Research Institute, National Institute of Advanced Industrial Science and Technology (AIST), AIST Tsukuba East, 1-2-1 Namiki, Tsukuba, Ibaraki 305-8564 Japan; 2 Mitsubishi Gas Chemical Trading, Inc., KANDA SQUARE 15F, 2-2-1 Kanda-Nishikicho, Chiyoda-Ku, Tokyo, 101-0054 Japan; 3 Sumitomo Bakelite Co., Ltd., 7-1 Satsukicho, Kanuma, Tochigi 322-0014 Japan

**Keywords:** Engineering, Optical materials and structures, Surface patterning

## Abstract

As society becomes smarter, advanced optical sensing and imaging technologies utilizing visible and near-infrared regions have become increasingly prevalent. Wire-grid polarizers, which are available for broadband electromagnetic waves, are effective in improving the signal-to-noise ratio of such optical systems and enabling more advanced object detection and analysis. However, to be implemented in everyday products, low-cost manufacturing methods must be developed while maintaining high-performance optical functions. To meet these requirements, we conducted an analysis of the geometry of wire-grid polarizers, and designed and developed a wire-grid polarizer with a nano-triangular wave-shaped structure that can be fabricated using general-purpose manufacturing equipment. Once the mould is prepared, this polarizer can be fabricated via nanoimprinting and metal deposition with a normal angle or electroless plating processes. The polarizer fabricated through electroless Ni plating achieves a transmittance of 40%, which is approximately 1.4 times higher than that achieved in a previous study using electroless Ni plating on a rectangular structure with the same period. In addition, the polarizer fabricated through normal angle Al deposition operates over a wide range of wavelengths from visible light to near-infrared, and achieves a polarization extinction ratio of 24 dB at a wavelength of 550 nm and a high transmittance of 81%. High-performance polarizers can be obtained through normal-angle deposition using general-purpose equipment in contrast to the oblique-angle deposition method employed in the manufacture of conventional rectangular structure-based wire-grid polarizers, thereby contributing to cost reduction and improved manufacturability.

## Introduction

Polarizers are important optical elements used in optical technologies, such as optical sensing and optical imaging; in the future, with the construction of a smart society, the demand for polarizers will increase further. Their applications using visible to near-infrared light are not limited to displays; in recent years, they have been used in light detection and ranging systems for automatic driving^1–4^, robots^5,6^, smartphones^7^, biological imaging systems^8–10^, and security systems^11,12^. As a result, research and development of polarizers has advanced significantly, leading to the emergence of metamaterial polarizers^13,14^, carbon nanotube polarizers^15–17^, and multilayer polarizers^18,19^, which have been proposed and demonstrated. Most current polarizers in the market are dichroic dye-based polarizers. Broadening the range of functional wavelengths is challenging, and the choice of polarizers in the near-infrared region is limited^20,21^. Wire-grid polarizers (WGPs) are promising candidates because they exhibit high performance over broadband wavelengths from the visible light to the near-infrared region by controlling the shape and material of the subwavelength anisotropic structure.

However, the high manufacturing cost of typical WGPs limits their application. To be adopted in various types of optical systems in the future, a low-cost manufacturing method is required. To fabricate metallic structures with the required subwavelengths, a more orthodox approach is to use electron beam lithography and Al etching. As alternative approaches, methods that use interference exposure^22^ and nanoimprinting^23,24^ instead of electron beam lithography have been reported. Nanoimprinting is a mould-based forming method that is relatively inexpensive among the nanostructure-forming methods. Subsequently, various nanoimprinting process-based methods that do not require Al etching, such as oblique-angle deposition^25–27^ and glancing angle deposition^28,29^, have been reported. As the removal of unnecessary metal portions is not required, the manufacturing process can be shortened and manufacturability can be improved. However, because special equipment for oblique-angle deposition is necessary and the required accuracy of the deposition angle is high, several WGPs using vacuum deposition at normal angles that can be manufactured using general-purpose equipment have been reported^30–35^. Furthermore, WGPs produced through solution processes that do not utilize the vacuum deposition method have also been reported^36–39^; nonetheless, their performance is inferior compared with that of WGPs produced through vacuum deposition. Thus, there is renewed interest in the research and development of low-cost, high-performance WGPs for implementation in next-generation applications. The realization of this technology is expected to make a significant contribution to the spread of advanced optical sensing and image processing systems and to realize a safer and more secure smart society.

In this study, we developed a high-performance nano-triangular wave-shaped polarizer (nano-TWP) via a fabrication process involving nanoimprinting and normal-angle Al vacuum deposition. This nano-TWP is suitable for low-cost metal thin-film formation methods, such as electroless Ni plating and normal-angle deposition, because the optical function is obtained by forming a nano-triangular wave structure on the substrate surface and metal film along the surface. The nano-triangular wave structure exhibits excellent mould release properties and enhances mould durability because it causes negligible friction in the mould release direction during the nanoimprinting process. Our research will enable engineers from a wide range of fields to fabricate prototype polarizers by simply preparing substrates with nano-triangular wave-shaped surfaces, thus facilitating the development of application products using polarizers. Therefore, the fabrication of nano-TWPs using electroless plating or vacuum deposition and the demonstration of their polarization function will have a significant impact not only on academia but also on the industry.

## Results and discussion

### Electroless Ni plating

**Figure 1:**
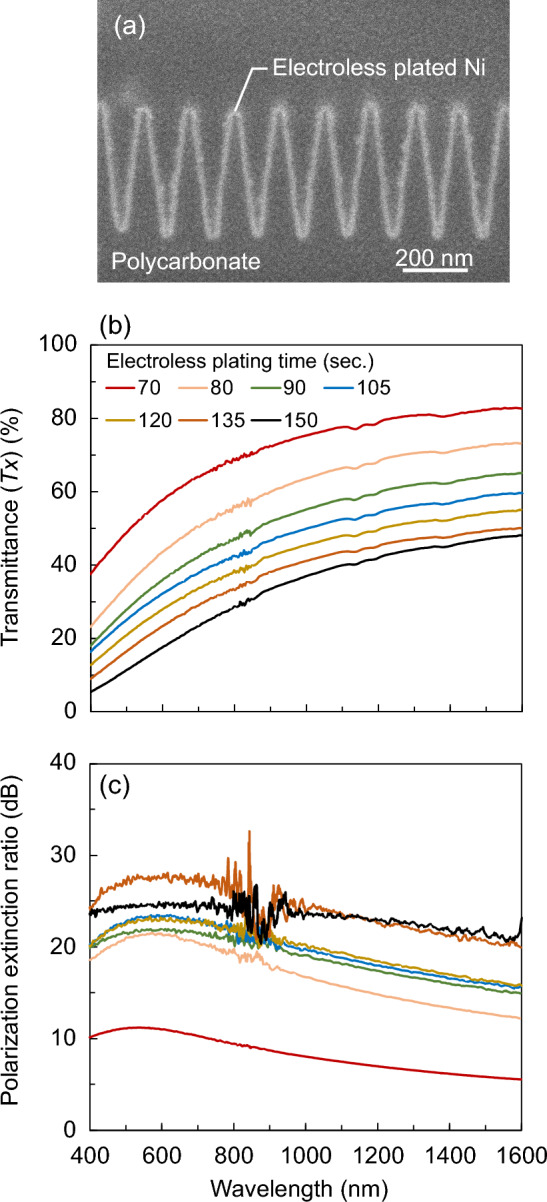
(**a**) Cross-sectional SEM image of the fabricated nano-TWP via electroless Ni plating. Measured (**b**) transmittance spectra and (**c**) PER spectra as a function of electroless plating time.

Electroless Ni plating is widely used in automotive and other industries; thus, conducting research to improve the polarizing properties using a low-cost liquid process is reasonable. Figure 1a shows the cross-sectional scanning electron microscopy (SEM) image of a nano-TWP fabricated via electroless Ni plating. As designed, a continuous triangular wave structure with almost no flat areas was formed on the surface of the polycarbonate sheet, and a Ni film was successfully formed uniformly on the surface of the polycarbonate sheet. In the nano-triangular wave structure, the duty cycle of the Ni portion in the layer at a certain position on the *z*-axis was almost constant from the tip to the bottom of the structure, and the average *D* value was 20%, as observed in Fig. 1a. Figure 1b,c show the transmittance at the *x*-polarized light incidence and polarization extinction ratio (PER) spectra measured using electroless plating time, respectively. Measurements were performed using a spectrophotometer (SolidSpec-3700, Shimadzu Corporation). The thickness of the Ni film can be controlled through the electroless plating time; the longer the plating time, the thicker is the film. Compared with the processing time, *Tx* decreased gradually from 54 to 14% as the processing time increased from 70 to 150 s at a wavelength of 550 nm, which is close to the peak sensitivity of the human eye. At a wavelength of 1550 nm, which is used in near-infrared sensing systems, *Tx* was 83% at a processing time of 70 s and 48% at a processing time of 150 s. The PER calculated from the measured *Tx* and *Ty* tended to increase as the processing time increased; however, it did not increase at a constant rate. At a wavelength of 550 nm, the PER was 13 dB at a processing time of 70 s, and the maximum PER of 27 dB was obtained at a processing time of 135 s. The PER at 1550 nm wavelength was lower than that at 550 nm at all treatment times: 7 dB at 70 s treatment time and 21 dB at 150 s treatment time. In comparison with a previous study^39^, electroless Ni plating on a rectangular structure with the same period showed a single transmittance (*Tx* + *Ty*)/2 of 14% at a similar PER at a wavelength of 550 nm, while for the nano-TWP, a single transmittance of 20%, approximately 1.4 times higher, was obtained at the treatment time of 80 s. Although the transmittance improvement rate was relatively low, the experimental results showed that the transmittance can be improved by using the nano-triangular wave structure compared with the conventional rectangular structure while maintaining a suitable PER in the production of nano-TWP via electroless Ni plating.

### Normal-angle Al deposition

**Figure 2:**
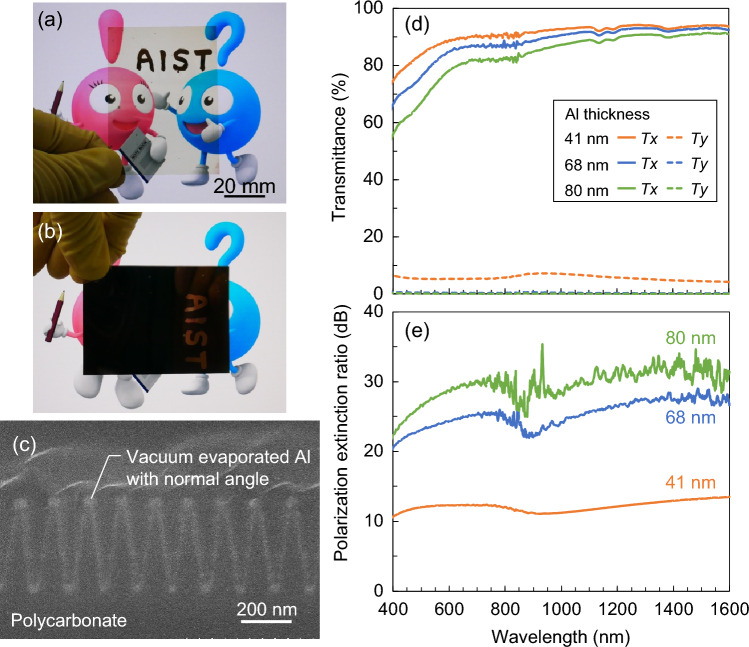
Photographs of the fabricated nano-TWP via normal-angle Al deposition arranged with (**a**) parallel and (**b**) crossed Nicols. (**c**) Cross-sectional SEM image of the fabricated sample. Measured (**d**) transmittance and (**e**) PER spectra as a function of Al thickness.

Al evaporation is used in several conventional WGPs in the visible and near-infrared regions. If a highly functional WGP can be realized using normal-angle deposition without applying the oblique-angle deposition method, which requires special equipment and precise angle control, it can significantly benefit the industry in terms of manufacturability and yield. Figure 2a,b show photographs of the fabricated nano-TWP held against the polarized image in parallel and crossed Nicols arrangements, respectively. Figure 2c shows the SEM image of the nano-TWP fabricated via normal-angle Al deposition. An Al thin film was formed on the entire surface of the triangular wave structure. In this case, the thickness of Al formed on the top of the structure was 41 nm, and the width of Al formed on the side wall of the inclined structure in the *x*-direction was approximately 17 nm on average. Therefore, the average duty cycle of the Al portion in the layer at a certain position on the *z*-axis was 24%, and it is possible to form *D* below a certain value from the tip to the bottom. Figure 2d,e show the measured transmittance and PER spectra, respectively, as a function of the thickness of the deposited Al film. In Fig. 2d, the solid and dashed lines plot *Tx* and *Ty*, respectively. The deposited Al film thicknesses were 41 nm, 68 nm, and 80 nm, and we confirmed that the average *D* increased according to the film thickness; the average *D* was 37% when the film thickness was 80 nm. The thinner the film, the higher is the single transmittance; the thicker the film, the higher is the PER. When the thickness of the film was 80 nm, a PER of 28 dB at a wavelength of 550 nm and that of approximately 30 dB in the near-infrared region was obtained. A suitable balance between the single transmittance and PER was obtained at a film thickness of 68 nm, with a single transmittance of 41% and PER of 24 dB at a wavelength of 550 nm as well as a single transmittance of 47% and PER of 27 dB at a wavelength of 1550 nm. A value close to the theoretical limit of 50% for the single transmittance of a normal polarizer, including the base substrate, was achieved. A comparison of the optical performance of the fabricated nano-TWP and conventional WGPs is summarized in Table 1. The following conventional WGPs are listed: those fabricated using expensive lithography and Al etching methods and using three different deposition methods based on nanoimprinting. Compared with WGPs with normal-angle deposition, a significant improvement in transmittance was observed by only changing the wave structure from rectangular to triangular, despite the fabrication method being the same. In addition, compared with WGPs with oblique-angle deposition, almost the same optical performance was obtained, although the PER was slightly inferior. The advantage of normal-angle deposition is that it can be implemented using general-purpose deposition equipment because the special stage mechanism and precise angle control required for oblique-angle deposition are unnecessary.

**Table 1:** Performance comparison between the fabricated nano-TWP and conventional WGPs.

Fabrication method	Single transmittance	PER	References
Lithography and etching	43–45%	> 30 dB	^15–17^
Nanoimprinting and oblique-angle deposition	40%	~ 30 dB	^18–20^
Nanoimprinting and glancing angle deposition	28%	15 dB	^22^
Nanoimprinting and normal-angle deposition	10–25%	~ 30 dB	^23–28^
Our study	41% 37%	24 dB 28 dB	

**Figure 3:**
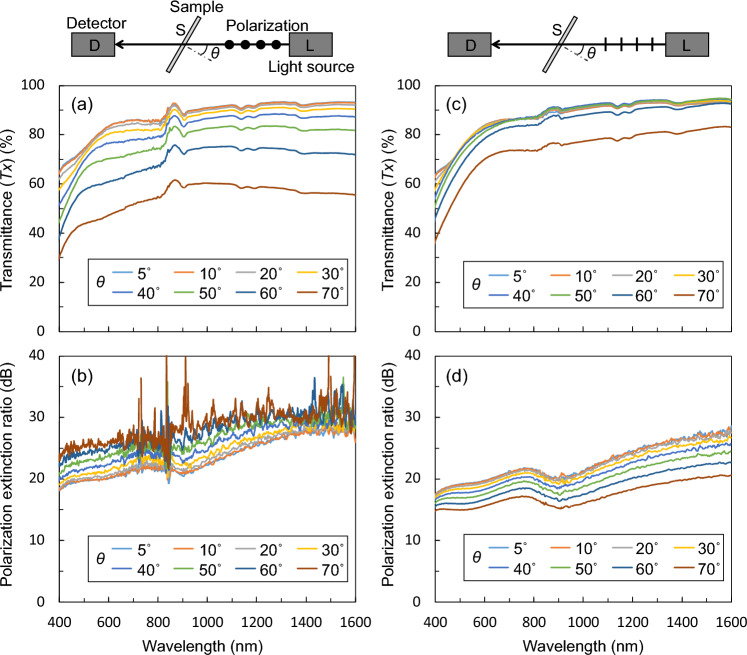
Incidence-angle dependence of the fabricated nano-TWP. Measured (**a**) transmittance and (**b**) PER spectra with the incident polarized light parallel to the rotation plane of the sample stage. Measured (**c**) transmission and (**d**) PER spectra with the incident polarized light parallel to the rotation plane of the sample stage.

From a practical perspective, we measured the incident-angle dependence of the nano-TWP and compared it with that of a conventional WGP. Figure 3a,b show the measured transmittance and PER spectra as a function of the incident angle *θ* of light when vertically polarized light is incident on the stage rotation plane. As *θ* increased, *Tx* decreased gradually to 45% at a wavelength of 550 nm for *θ* = 70°. Conversely, the PER increased gradually; at a wavelength of 550 nm, a PER of 20 dB was obtained for *θ* = 5°, whereas a PER of 25 dB was obtained for *θ* = 70°. Figure 3c,d show the measured transmittance and PER spectra when the polarized light parallel to the rotation plane of the sample stage is incident. *Tx* was insensitive to changes in *θ*, and there was no significant change in the *Tx* spectra up to approximately *θ* = 50°. However, the PER gradually decreased, and at a wavelength of 550 nm, the PER decreased from 19 dB when *θ* = 5° to 15 dB when *θ* = 70°. Compared with the incident-angle dependence of conventional WGPs, we confirmed that the tendency of nano-TWP is almost the same^40,41^. Therefore, the influence of the change from conventional WGP to nano-TWP on the incident-angle dependence is small.

**Figure 4:**
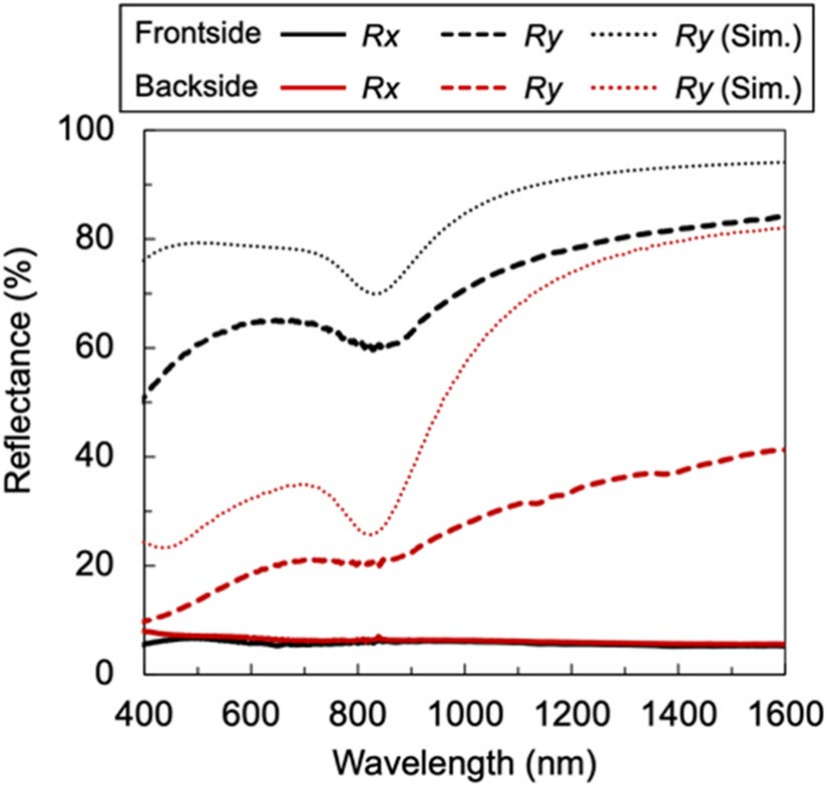
Measured reflectance spectra of the front and back sides of the fabricated nano-TWP. Dotted lines show the calculated plots.

Next, the reflectance spectra were evaluated to determine their applicability as reflective or absorptive polarizers. Figure 4 shows the measured and calculated reflectance spectra. Reflectance was measured as a normal reflection at *θ* = 5°. Measurements incident on the structural surface side and on the back side were plotted as the front and back side results, respectively. These measurements were the total light reflected by the sample and not the reflectance results for the topmost surface of only one side. Notably, there was a marked difference in reflectance between the front and back sides. For the front side, the reflectance was similar to that of a reflective polarizer, whereas for the back side, as shown in Fig. 2b, it appeared dark, similar to an absorptive polarizer. Using the shape of the structure in the SEM image as a model, numerical calculations were conducted via rigorous coupled-wave analysis (RCWA)^42^. The refractive index of Al was obtained from the study by Rakić et al.^43^ The calculated values for both the frontside and backside reflectance were higher than the experimental values; nonetheless, the trends were in a relatively good agreement. A spectral haze meter (SH-7000, Nippon Denshoku Industries) for visible light was used to evaluate the percentage of scattered light among the transmitted light. A haze of less than 1.0% was obtained in the visible region, indicating that the scattering component was small. Therefore, components other than the transmitted and reflected light relative to the incident light are almost entirely absorbed. Accordingly, the front side can be used as a reflective polarizer, while the back side can be used as a polarizer close to an absorptive type.

## Conclusion

In summary, we fabricated a WGP for the visible to near-infrared region based on a nano-triangular wave-shaped structure and evaluated its optical properties. The polarizers were fabricated via thermal nanoimprinting and metal deposition using either a normal angle or electroless plating processes. The polarizer fabricated through Ni electroless plating achieved a transmittance of 40%, which is approximately 1.4 times higher than that achieved in a previous study using electroless Ni plating on a rectangular structure with the same period. This result was obtained at a wavelength of 550 nm, while maintaining the same polarization extinction ratio. In addition, a high-performance polarizer to be realized by Al deposition at normal angles using general-purpose equipment, without using oblique-angle deposition that requires a special stage mechanism; a high transmission rate of 81% was obtained with a PER of 24 dB at a wavelength of 550 nm. These polarizers can be manufactured through a low-cost process by preparing a mould with a nano-triangular wave-shaped surface. This manufacturing method can be conveniently applied in industrial applications because it does not require any special equipment or complex process. In the future, we will clarify the correlation between optical properties, including the reflectance, and the nano-triangular wave-shaped structure to optimize optical functions to meet the social needs. We expect that the applications of low-cost and high-performance polarizers based on the findings in this study will accelerate and facilitate the realization of smart sensing and a digital society in the future.

## Design and fabrication

**Figure 5:**
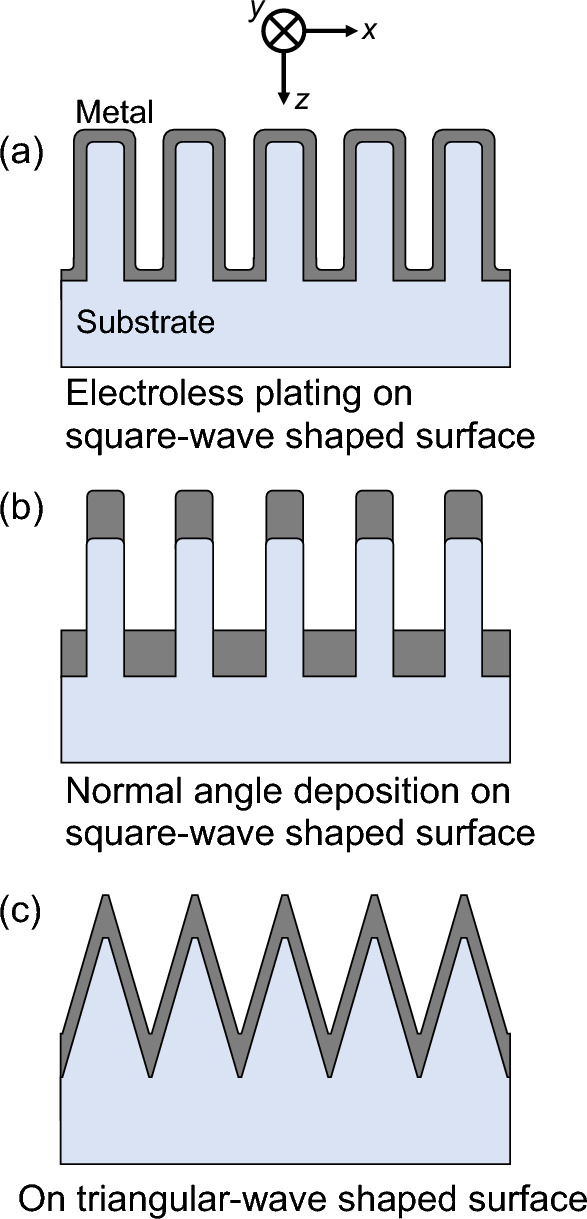
Illustration of (**a**) electroless plating on a rectangular surface, (**b**) normal-angle deposition on a rectangular surface, and (**c**) electroless plating or normal-angle deposition on a triangular-wave-shaped surface.

The significance of using a nano-triangular wave-shaped surface is explained by comparing it with a conventional rectangular wave-shaped surface. Figure 5a,b illustrate electroless plating and normal-angle deposition on a rectangular surface, respectively. In general, electroless plating, which is a low-cost manufacturing technique compared with evaporation, can form metal films along the surface. For normal-angle deposition, metal films are less likely to form on the sidewalls because the metal vapor flux is deposited linearly. In a certain layer of the structural part, the metal duty cycle *D* (the ratio of the metal part per period of the nanostructure) varies depending on its position along the z-axis. In Fig. 5a, *D* exceeds 50% in the layers at convex or concave bottoms. Even if the width of the convex portion is narrower, a higher *D* exceeding 50% appears in the layer along the z-axis because *D* at the bottom of the concave portion is larger. Similarly, in Fig. 5b, regardless of the extent to which the widths of the convex and concave portions are adjusted, layers with *D* exceeding 50% appear. Reducing *D* can effectively increase the optical transmittance of a WGP^44^. According to the effective medium theory, the effective refractive index in such subwavelength structures can be approximated using *D*^45^. In other words, the presence of a layer with *D* greater than 50% hinders the realization of high transmittance because the optical response in that layer is more similar to that of a metal than a dielectric. However, when electroless plating or normal-angle deposition is performed on the surface of the triangular wave shape, a metal film is formed, as shown in Fig. 5c. By controlling the thickness of the metallic film, *D* can be maintained below a certain value of smaller than 50% for any layer from the convex to the concave bottom, and high transmittance can be obtained while maintaining a high PER.

**Figure 6:**
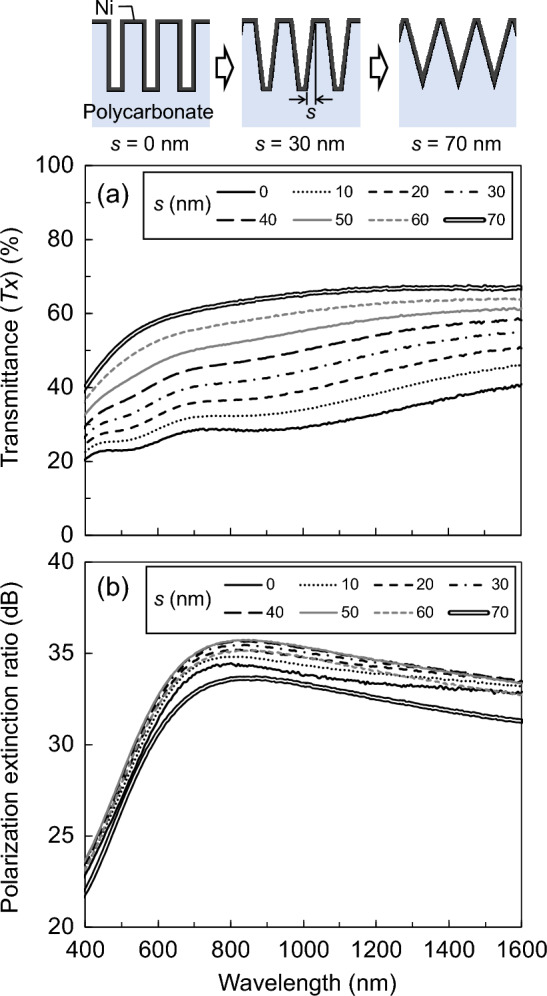
Calculated (**a**) transmittance spectra at x-polarization incidence and (**b**) the PER spectra.

Numerical calculations via the RCWA were performed to estimate the optical characteristics of nano-TWP. The upper inset of Fig. 6 shows the calculation model. The plastic substrate was polycarbonate with a thickness of 300 μm, and a concavo-convex structure with a height of 400 nm and a period of 140 nm was formed on its surface. Assuming that the metal film was formed by electroless Ni plating, a Ni film with a thickness of 10 nm was formed on the entire surface of the structure, including the sidewalls. The width from the edge of the convex to the edge of the concave bottom is denoted as s. As s changed from 0 mm to 70 nm, the structure changed from a rectangular shape to a triangular wave shape. The refractive index of polycarbonate was determined to be 1.58 and that of Ni was obtained from the study by Rakić et al.^43^ The tapered portion was divided approximately every 40 nm, which corresponds to 1/10th of the height of the concavo-convex structure, to determine the refractive index distribution of the layer. Figure 6a,b show the transmittance spectra at *x*-polarization incidence and the PER spectra as a function of s, respectively. PER was obtained using the following equation:

1 $$\mathrm{PER}=10{\mathrm{log}}_{10}\left(Tx/Ty\right),$$

where *Tx* and *Ty* are the transmittances at *x*- and *y*-polarization incidence, respectively. Figure 6a shows that the transmittance drastically increases as the structure is transformed from a rectangular shape to a triangular wave shape; that is, as the *D* between the convex and concave bottom of the structure gradually decreases, the transmittance increases from 24 to 55% at a 550-nm wavelength. Furthermore, Fig. 6b shows that the PER is insensitive to s. At a wavelength of 550 nm, PER was 29.3 dB when s = 0 nm, and at s = 70 nm, PER did not decrease considerably to 28.8 dB, maintaining a high PER. Therefore, the simulation confirmed that the nano-TWP exhibited improved transmittance while maintaining the PER.

**Figure 7:**
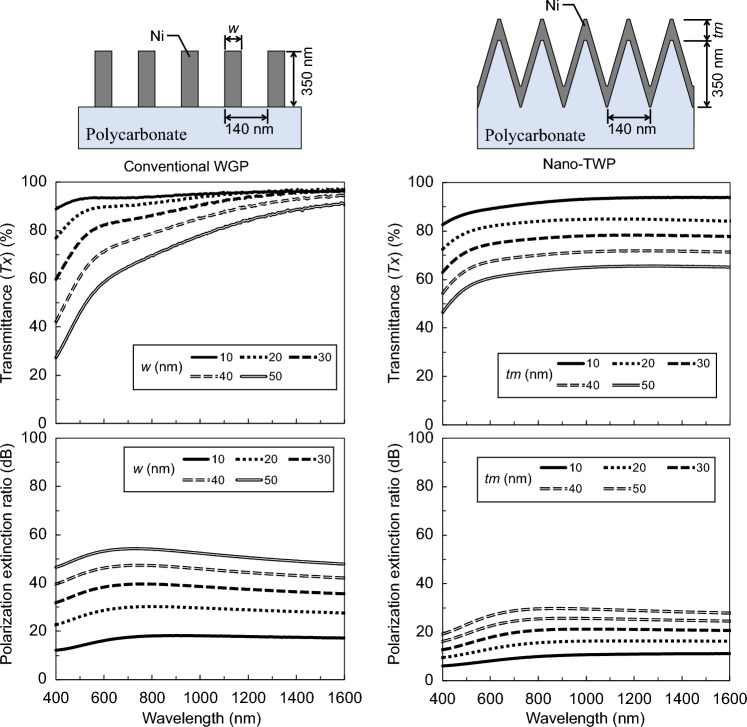
Calculated transmittance spectra at x-polarization incidence and PER spectra of a conventional line-and-space typed WGP and the nano-TWP.

To gain further insight into the nano-TWP, we compare it with line-and-space structural WGPs found in previous studies^15–17^ and WGPs fabricated using oblique angle deposition^18–20^. Given that the nano-TWP is a subwavelength structure, its composition is nearly identical to that of the conventional line-and-space. Specifically, when the cross-section of each structure is cut by a certain layer, the metal part and the air part are present in a particular occupancy ratio in the line-and-space structure. In the structure fabricated using the oblique angle deposition method, the metal part, the substrate material part, and the air part are present in a particular occupancy ratio. In the nano-triangular wave-shaped structure, the metal part, the substrate material part, and the air part exist, the ratio of the metal portion is almost constant from the surface to the bottom of the structure, while the ratio of the substrate portion gradually increases. Given that the configurations of these structures are almost identical, their optical characteristics are expected to be similar despite differing in shape. To confirm this assumption, a comparison was conducted using the conventional line-and-space structure through RCWA simulation. Figure 7 shows the calculation results when Ni was used as metallic materials. The refractive indices of polycarbonate and Ni were the same as those described above. In the case of nano-TWP, assuming uniform formation of Ni across the structure surface, an increase in Ni thickness in the *z*-direction (tm) not only increases the thickness of the metal-containing layer but also increases the thickness of the Ni film in the *x*-direction. Consequently, the ratio of the metal portion increases. Therefore, with an increase in tm, the PER increases while the transmittance decreases.

The calculation results show that both high transmittance and PER above 20 dB were also obtained in the nano-TWP, depending on the conditions. Compared with a conventional WGP, the transmittance was lower under the conditions necessary to achieve the same PER. However, PER can be increased without making *tm* too thick by increasing the height of the nano-triangular wave-shaped surface structure of the substrate, thereby improving polarization properties. Further investigation into the relationship between geometry and optical properties will be conducted in the future.

**Figure 8:**
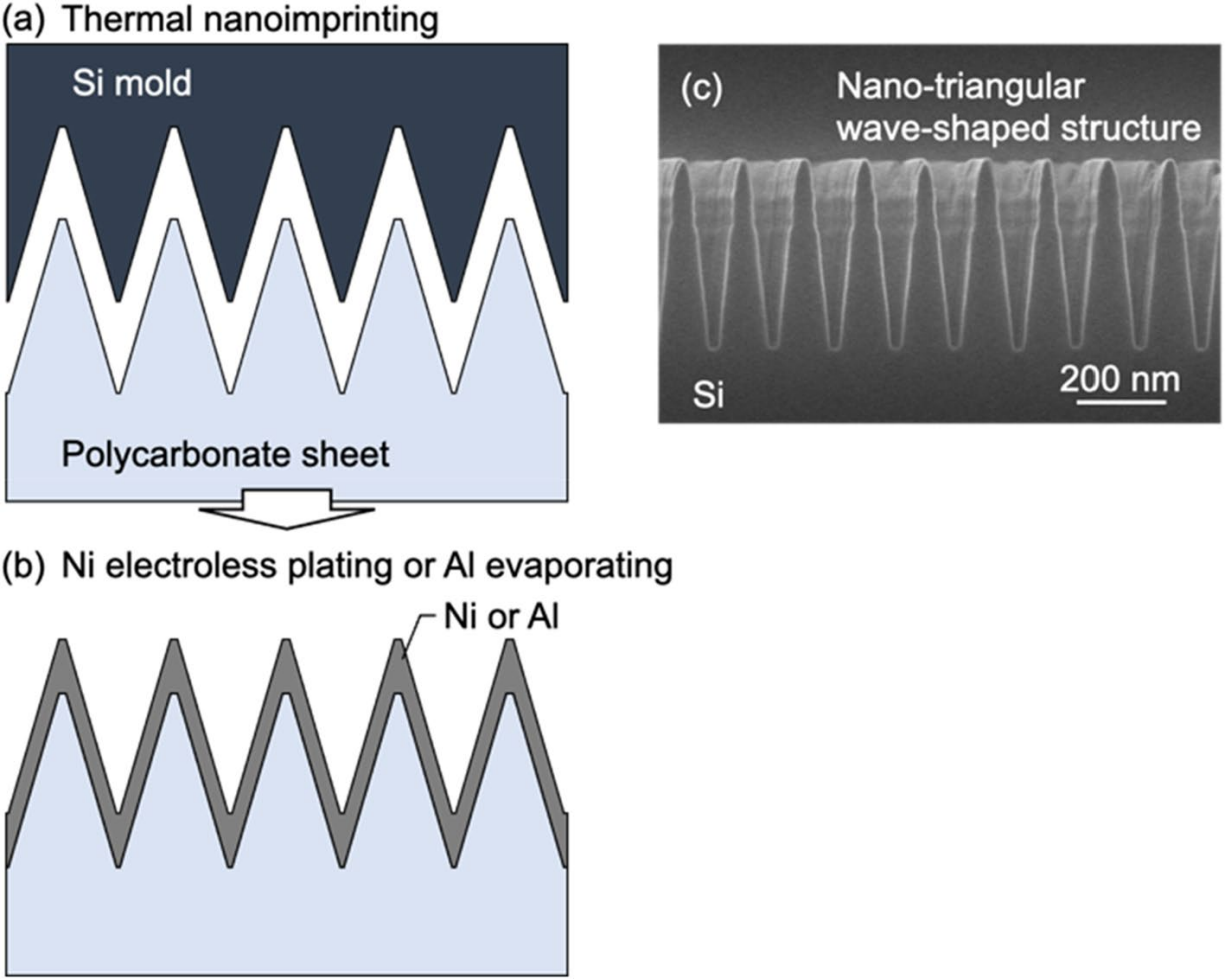
Fabrication process illustrations for (**a**) thermal nanoimprinting and (**b**) Ni electroless or Al evaporating; and (**c**) Cross-sectional SEM image of the mould.

Figure 8a,b show the nano-TWP fabrication process. In thermal nanoimprinting, the mould temperature was 170 °C, and a pressure of 0.9 MPa was applied for 150 s. Figure 8c shows a cross-sectional SEM image of the mould. The mould featured a nano-triangular wave-shaped structure with a period of approximately 140 nm and a height of approximately 420 nm; the mould was formed using lithography and reactive ion etching. After the thermal nanoimprinting step, the surface structure of the polycarbonate sheet shrunk slightly compared with that of the mould structure owing to thermal contraction. Next, by performing electroless Ni plating or Al deposition on the surface of the structure, nano-TWP was obtained. For electroless Ni plating, after being dipped into the sensitizer and activator solutions manufactured by Okuno Chemical Industries, the substrates were dipped into an electroless Ni plating bath manufactured by Okuno Chemical Industries at room temperature (23 °C). Al was deposited using an electron beam evaporation system at a normal angle perpendicular to the polycarbonate sheet surface. Electroless plating and vacuum deposition are typical manufacturing techniques for thin-film formation, and several companies already have this equipment.

## Data Availability

The data that support the findings of this study are available from the corresponding author upon reasonable request.
